# Avatrombopag for the Treatment of Immune Thrombocytopenia

**DOI:** 10.1111/ejh.14395

**Published:** 2025-02-04

**Authors:** Caterina Labanca, Ernesto Vigna, Enrica Antonia Martino, Antonella Bruzzese, Francesco Mendicino, Giulio Caridà, Eugenio Lucia, Virginia Olivito, Noemi Puccio, Antonino Neri, Fortunato Morabito, Massimo Gentile

**Affiliations:** ^1^ Hematology Unit Azienda Ospedaliera Annunziata Cosenza Italy; ^2^ Laboratorio di Ricerca Traslazionale Azienda USL‐IRCSS Reggio Emilia Reggio Emilia Emilia‐Romagna Italy; ^3^ Scientific Directorate IRCCS of Reggio Emilia Reggio Emilia Emilia‑Romagna Italy; ^4^ Gruppo Amici Dell'Ematologia Foundation‐GrADE Reggio Emilia Italy; ^5^ Department of Pharmacy, Health and Nutritional Science University of Calabria Rende Italy

**Keywords:** avatrombopag, ITP, therapy, TPO mimetics, TPO‐R

## Abstract

Avatrombopag, a second‐generation thrombopoietin receptor agonist (TPO‐RA), represents a significant advancement in the treatment of chronic immune thrombocytopenic purpura (cITP) and a potential therapeutic option for other thrombocytopenic disorders. Approved in both the USA and Europe, avatrombopag offers a convenient oral dosing regimen, initiated at 20 mg daily with food, to achieve and maintain platelet counts ≥ 50 × 10^9^/L. Its favorable safety profile, characterized by minimal hepatic toxicity and the absence of dietary restrictions, distinguishes it from older TPO‐RAs such as eltrombopag and romiplostim. Clinical trials and real‐world data support its efficacy, with over 90% of patients that fail to standard first‐ and second‐line treatments or become unresponsive, achieving target platelet counts, and its hepatotoxicity‐free profile makes it particularly advantageous for patients with liver disease or complex comorbidities. Economic evaluations, including a budget impact analysis for the Italian National Health Service, have projected significant healthcare cost savings associated with avatrombopag use, reinforcing its value as a cost‐effective therapeutic option. However, challenges remain, including limited data on long‐term safety. In this review, we aim to provide a comprehensive synthesis of clinical evidence and real‐world data on avatrombopag's efficacy, safety, and pharmacological advantages, while exploring its current and potential therapeutic applications, such as chemotherapy‐induced thrombocytopenia and aplastic anemia.

## 1 Introduction

Thrombocytopenia is defined as a platelet count below 150 × 10^9/L, predisposing individuals to an increased risk of bleeding and, in severe cases, life‐threatening hemorrhages, particularly when counts fall below 30 × 10^9/L. This condition is especially concerning in older adults over 60 years of age where frailty amplifies these risks. Common etiologies include chronic liver disease, pregnancy, chemotherapy, aplastic anemia, and viral infections [1, 2, 3].

Immune thrombocytopenia (ITP) is an acquired autoimmune disorder characterized by a platelet count below 100 × 10^9/L, accompanied by normal white blood cell and hemoglobin levels. According to the American Society of Hematology (ASH), ITP presents clinically with symptoms such as purpura or other signs of bleeding. Its incidence ranges from 1 to 6 cases per 100 000 people annually [4, 5], with a predominance among young women and older men. Recent trends indicate an increasing incidence, possibly due to improved recognition and diagnostic practices [4, 6, 7]. Approximately 80% of ITPs are idiopathic, while the remaining are associated with underlying conditions such as autoimmune diseases, viral infections, drugs, and vaccinations [6, 8, 9].

The pathophysiology of ITP involves platelet destruction mediated by autoantibodies, primarily IgG, that target platelet membrane glycoproteins (GP IIb/IIIa, Ib/IIa, and VI). These autoantibodies accelerate platelet clearance by macrophages, predominantly in the spleen, while also impairing platelet production. Emerging evidence suggests that T‐cell‐mediated cytotoxicity may also contribute to megakaryocyte damage in the bone marrow, further reducing platelet production [10, 11].

Clinically, patients with ITP may present with bleeding manifestations ranging from petechiae and ecchymoses to severe visceral or intracranial hemorrhages. In 2009, the International Working Group (IWG) established standardized definitions for the classification of ITP into three phases: *newly diagnosed* (duration < 3 months), *persistent* (3–12 months), and *chronic* (> 12 months) [12, 13, 14].

The 2019 ASH guidelines and the International Consensus Report recommend initiating treatment in patients with platelet counts below 20–30 × 10^9/L to mitigate bleeding risks and improve quality of life [12, 13]. Treatment response is defined by a platelet count of 30–50 × 10^9/L or a doubling from baseline, while complete response (CR) is achieved with counts exceeding 100 × 10^9/L [12, 13].

In acute severe ITP with bleeding, platelet transfusion remains the primary emergency intervention. However, its utility is limited by risks such as transfusion reactions and antiplatelet antibody formation [12, 13, 15]. First‐line therapies include corticosteroids, intravenous immunoglobulin (IVIG), and intravenous anti‐D immunoglobulin, achieving a response in approximately 75% of patients within 7–10 days [11, 12, 13, 14, 15, 16, 17]. Yet, 30%–40% of patients may exhibit corticosteroid resistance or intolerance.

Second‐line treatments include agents such as dexamethasone at high‐dose, danazol, dapsone, immunosuppressants, and the monoclonal antibody rituximab. Splenectomy, once a mainstay of second‐line treatment, is now less favored due to its invasive nature, long‐term risks, and the potential for relapse [12, 13, 15]. While these treatments have shown promise, their efficacy, adverse effects, and cost‐effectiveness remain underexplored due to a lack of randomized clinical trials.

The discovery of thrombopoietin (TPO) and its receptor (TPO‐R, c‐Mpl) in the 1990s revolutionized ITP treatment. TPO, a hematopoietic growth factor, binds to TPO‐R, activating signaling pathways including Janus kinase (JAK)/signal transducer and activator of transcription (STAT), mitogen‐activated protein kinase (MAPK), and phosphatidylinositol‐3 kinase (PI3K). These pathways promote megakaryocyte precursors and megakaryocyte maturation, platelet production, and anti‐apoptotic effects [18, 19, 20, 21, 22, 23, 24, 25].

Based on these insights, the advent of platelet‐stimulating agents, such as TPO and thrombopoietin receptor agonists (TPO‐RAs), has shifted significantly the paradigm of chronic ITP (cITP) treatment [12, 13, 14, 15, 26]. Initial therapeutic efforts with recombinant human TPO (rhTPO) and pegylated recombinant human megakaryocyte growth and development factor (PEG‐rHuMGDF) were halted in 1998 due to the induction of autoantibodies cross‐reacting with endogenous TPO. Subsequently, second‐generation TPO‐RAs were developed to mimic the structure of endogenous TPO while avoiding immunogenicity [27, 28].

Currently, the TPO‐RAs—romiplostim, eltrombopag, avatrombopag, and lusutrombopag—have specific indications approved by the US Food and Drug Administration (FDA) and the European Medicines Agency (EMA). The peptibody romiplostim competes with endogenous TPO for receptor binding [29, 30, 31, 32, 33, 34], whereas small‐molecule agonists interact with a distinct transmembrane receptor domain. These agents have demonstrated efficacy not only in cITP but also in conditions such as chronic liver disease, aplastic anemia, and hepatitis C [29, 30, 31, 32, 33, 34, 35, 36, 37, 38, 39, 40, 41, 42]. The ongoing development of new agents, such as hetrombopag, reflects the continuous evolution of cITP management, addressing gaps in long‐term efficacy, safety, and expanded indications [43].

The purpose of this review is to provide a comprehensive overview of avatrombopag, highlighting its pharmacological characteristics, clinical efficacy, safety profile, and therapeutic potential in the management of cITP.

## 2 Mechanism of Action, Pharmacodynamic, and Pharmacokinetic

Avatrombopag, originally identified as YM477 and AKR 501, represents the most recently approved TPO‐RA receiving FDA approval in June 2019 for the treatment of adult patients with cITP who have demonstrated an inadequate response to previous therapies. Clinical studies have shown its efficacy to be comparable to that of other available therapeutic options [37, 38, 39, 40, 41, 42, 43, 44, 45].

Avatrombopag is a small molecule agonist of the TPO‐RA that increases platelet production by activating the receptor, mimicking the biological actions of TPO [46]. Its unique chemical structure enables selective binding to the transmembrane domain of the TPO‐R, avoiding interference with endogenous TPO binding. This specificity, attributable to a histidine residue at position 499, makes avatrombopag effective and species‐specific, particularly in humans and chimpanzees [46]. In vitro studies demonstrate that avatrombopag stimulates the proliferation of murine Ba/F3 cells expressing human TPO‐R in a concentration‐dependent manner (EC50 3.3 nmol/L), achieving maximal activity comparable to rhTPO. Cells lacking TPO‐R showed no response to avatrombopag, confirming receptor‐dependent activity [46].

**FIGURE 1:**
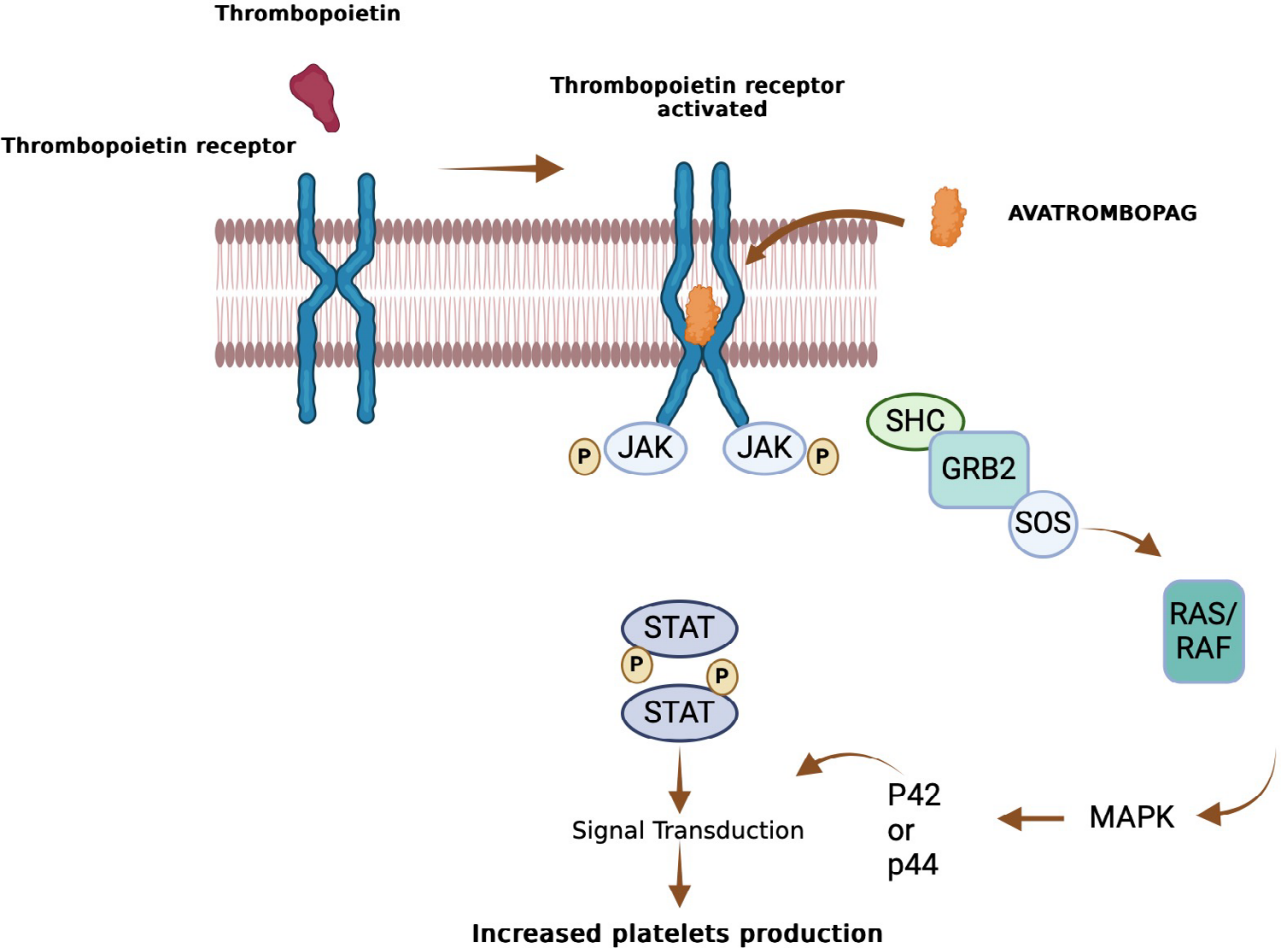
Mechanism of action of avatrombopag. Avatrombopag selectively binds to the transmembrane domain of the TPO receptor without interfering with TPO's binding. It promotes the tyrosine phosphorylation of STAT3 and STAT5, along with threonine phosphorylation of the MAPK (ERK) pathway, resulting in significant differentiation and proliferation of megakaryocytes, essential for platelet generation.

Further studies indicated that avatrombopag activates key intracellular pathways, including the phosphorylation of STAT3 and STAT5, and ERK (MAPK), promoting differentiation and proliferation of megakaryocytes, essential for platelet production (Figure 1).

Avatrombopag synergistically enhances megakaryocyte colony formation, mimicking rhTPO‐induced platelet maturation. In studies using human hematopoietic CD34+ cells, avatrombopag stimulated megakaryocyte differentiation in a concentration‐dependent manner (EC50 25.0 nmol/L) with maximal activity akin to rhTPO. Notably, in cultures using G‐CSF–mobilized human peripheral blood CD34+ cells treated with both avatrombopag and rhTPO, megakaryocyte proliferation increased by 200%, indicating an additive effect during early maturation stages. Importantly, avatrombopag did not affect the binding of radiolabeled rhTPO to the TPO receptor, suggesting that it interacts with a different site than the natural ligand [46].

In vivo studies in non‐obese diabetic/severe combined immunodeficiency mice, which were transplanted with human fetal liver CD34+ cells, demonstrated that oral avatrombopag administration led to a dose‐dependent increase in human platelet counts, without affecting platelet activation or reactivity levels, emphasizing its safety and specificity [47].

Pharmacokinetic (PK) assessments revealed that avatrombopag is absorbed slowly after oral administration, with a lag time of 0.5–0.75 h and peak plasma concentrations at 6–8 h. Steady‐state levels are achieved by day 5 in multiple‐dose regimens. The drug exhibited dose‐proportional pharmacokinetics up to 80 mg, and food co‐administration reduced variability (Cmax and AUC) by approximately 50%, though it does not significantly alter these parameters [48]. Moreover, avatrombopag demonstrates high plasma protein binding (> 96%) and a large volume of distribution (~235 L in ITP patients, ~180 L in those with liver disease‐associated thrombocytopenia), suggesting extensive tissue distribution [48]. Metabolism occurs primarily via cytochrome P450 isoenzymes CYP2C9 and CYP3A, with fecal excretion accounting for 88% of drug elimination. Plasma does not contain detectable metabolites and the terminal half‐life is approximately 19 h [48]. Drug interaction studies revealed that co‐administration with fluconazole, a dual CYP2C9 and CYP3A inhibitor, doubled avatrombopag's AUC and half‐life, resulting in a clinically relevant 1.66‐fold increase in platelet counts. In contrast, CYP3A inhibitors alone (e.g., itraconazole) had minimal effects, indicating CYP2C9 predominance in avatrombopag metabolism. Conversely, coadministration of avatrombopag with rifampin, a CYP2C9 and CYP3A inducer, halved AUC and half‐life of avatrombopag, but did not significantly affect platelet counts [47].

Based on data from phase I to III studies involving 787 patients and volunteers, PK modeling identified a one‐compartment model with simultaneous first‐ and zero‐order absorption and linear elimination factors such as body weight and the presence of chronic liver disease markedly influenced the apparent volume of distribution, while East Asian ethnicity, albumin level, and TPO levels affected platelet response without clinical relevance [49].

Avatrombopag consistently induces a dose‐dependent increase in platelet counts in patients with ITP and liver disease‐associated thrombocytopenia, maintaining normal platelet activation levels. This unique mechanism enhances platelet production without increasing reactivity, distinguishing it as a safer option [50]. Phase II and III clinical trials confirmed its favorable safety profile, including no significant QT interval prolongation [51, 52, 53, 54]. Its pharmacodynamic (PD) and PK characteristics make avatrombopag a promising treatment option for managing thrombocytopenia across diverse clinical populations, culminating in its FDA approval following successful clinical trials.

## 3 Clinical Trials

### 3.1 Pivotal Trials

The clinical development of avatrombopag has been underpinned by a series of carefully designed trials (Table 1), beginning with Phase I studies that explored its safety, tolerability, pharmacokinetics, and effects on platelet counts in healthy adults. These randomized, double‐blind, placebo‐controlled trials laid the groundwork for subsequent research by demonstrating both the biological activity and tolerability of avatrombopag. The single‐dose study included 63 participants, randomized in a 2:1 ratio to receive various doses of avatrombopag (1, 3, 10, 20, 50, 75, and 100 mg) or a placebo. Meanwhile, the multiple‐dose study involved 29 subjects who received daily dose (3, 10, or 20 mg) over two weeks. Across both trials, avatrombopag exhibited a favorable safety profile, with no serious AEs, dose‐limiting toxicities, or abnormalities in liver function reported. Pharmacokinetic evaluations revealed dose‐proportional increases in drug exposure and consistent half‐life values (18–21 h), supporting a once‐daily dosing regimen. Platelet count responses were contingent upon the dose, concentration, and duration of treatment. Increases in platelet counts were observed beginning 3 to 5 days after administration, with a maximum increase exceeding 370 × 10^9/l above baseline seen with a daily dose of 20 mg after 13 to 16 days. These compelling findings support the continued development of avatrombopag for the management of various thrombocytopenic conditions and provide important guidance for hematologists in implementing this new thrombopoietin receptor agonist within clinical practice [51].

**TABLE 1:** Pivotal trials.

Clinical trial ID	Phase	Patients enrolled (*n*)	Efficacy	Safety (AEs)
NCT00441090 (Study 003) [51]	II Randomized study 28 days	Total (*n* = 64) Placebo *n* = 5 2.5 mg *n* = 15 5 mg *n* = 15 10 mg *n* = 14 20 mg *n* = 15	Placebo = 0% 2.5 mg = 13% 5 mg = 53% 10 mg = 50% 20 mg = 80%	Any grade AE = 100% (64/64) Severe AEs = 41% (26/64) Serious AEs = 19% (12/64)
NCT00625443 (Study 004) [51]	II Extension part 24 weeks	Total (*n* = 53) Responders *n* = 25 Non Responders *n* = 28	Overall Response: 76% Durable Response: 53% 3/24 reduced steroid doses by ≥ 50% 8/24 discontinued steroids	—
NCT01438840 (Amendment 02 trial) [52]	III Core study (6 months)	Total (*n* = 49) Avatrombopag *n* = 32 Placebo *n* = 17	Duration of PCs ≥ 50 × 10^9^/L (Avatrombopag versus Placebo) Median 12.4 versus 0.0 weeks Mean 12.0 versus 0.1 weeks Durable platelet RR 34.4% versus 0%	TEAEs 96.9% vs. 58.8 Headache, contusion, upper respiratory tract infection, arthralgia, epistaxis, fatigue, gingival bleeding petechiae, thrombocytopenia, pharyngitis, hypertension, nasopharyngitis SAEs 28.1 versus 5.9% Headache Vomiting Platelet count decreased
	III Extension phase (90 weeks)	Total (*n* = 39)	Response rate 96.1% Complete response rate 60.1%	Core ± extension phase Avatrombopag incidence (*n* = 47) TEAEs 95.7% SAEs 31.9%

Abbreviations: AEs: adverse events; n: number of patients; SAEs: serious adverse event; TEAEs: treatment‐emergent adverse events.

Avatrombopag's efficacy in ITP was further demonstrated through a pivotal Phase II trial and a Phase III trial, which formed the basis of its FDA approval for adults with chronic ITP unresponsive to other treatments [52, 53].

The Phase II trial (NCT00441090) was a multicenter, double‐blind, randomized, placebo‐controlled, dose‐ranging, parallel‐group study investigating the avatrombopag PK and PD relationship in 65 patients with ITP lasting at least three months. Participants were randomized into five treatment groups (2.5, 5, 10, or 20 mg or placebo for 28 days), in a 3:3:3:3:1 ratio. Participants were randomized into five treatment groups (2.5–20 mg daily or placebo) and monitored for safety and efficacy. Platelet response rates, defined as achieving counts ≥ 50 × 10^9^/L with an increase of at least 20 × 10^9^/L, improved with increasing doses. Remarkably, 80% of participants in the 20 mg group achieved a response compared to none in the placebo group. Responses were rapid, with 93% of patients in the highest‐dose group responding by day 7. A subsequent 24‐week extension phase revealed durable responses in over half of the participants, with 76% achieving an overall response and many able to reduce or discontinue corticosteroid therapy. This extension phase highlighted the sustainability of avatrombopag's effects and its potential role as a long‐term treatment option [52].

After the study period on Visit Day 28 ± 1, participants who had completed 28 ± 1 days of dosing were assessed for their eligibility to transition into the subsequent rollover study designated as NCT00625443 (Study 501‐CL‐004). This assessment was conducted as part of a planned continuation to provide further insight into the long‐term efficacy and safety of avatrombopag in a controlled setting.

The NCT01438840 (Amendment 02 trial) was a phase III, multicenter trial that included three phases: pre‐randomization, a 26‐week Core Study, and a 76‐week Extension Study, totaling about 104 weeks of treatment. According to enrolment criteria, patients included in the study were diagnosed with cITP for at least 12 months, with an average platelet count > 30 × 10^9/L and no alternative causes of thrombocytopenia. Eligible patients had previously been treated for ITP with corticosteroids or rituximab and showed a prior response to ITP therapy (platelet count > 50 × 10^9/L) or had a bone marrow examination consistent with ITP within the last 3 years to exclude other hematological conditions. Additionally, participants exhibited PT/INR and aPTT values within 80%–120% of the normal range, with no evidence of hypercoagulable states.

A total of 49 participants were enrolled in the study and randomized in a 2:1 ratio to receive either avatrombopag (*n* = 32) or placebo (*n* = 17), with subsequent dose adjustments based on individual platelet response. This study utilized a dose‐titration approach to maintain platelet counts within a target range of 50–150 × 10^9/L while reducing reliance on rescue therapies. The trial's primary endpoint, that is the cumulative number of weeks with platelet counts ≥ 50 × 10^9^/L without rescue therapy, significantly favored avatrombopag (median 12 weeks versus 0.1 weeks for placebo, *p* < 0.0001). Additionally, 66.8% of patients in the avatrombopag group achieved a rapid platelet response by day 8, and 33% experienced reductions in concurrent ITP treatments. Notably, the platelet counts in the treated group peaked at day 14, likely as a result of a “first dose effect” from avatrombopag. Sustained responses were observed throughout the core 26‐week study and into the 76‐week extension, with a durable response rate (≥ 6 of the last 8 weeks of treatment) of 34.4% in avatrombopag‐treated patients versus 0% for placebo (*p* = 0.009) and a median of 12.4 cumulative weeks of platelet response during the core study (24 weeks) [55]. Post hoc analyses confirmed that response rates remained high during extension phase visits, with rates of 96.1% of durable responders and 60.1% of complete responders (defined as platelet count ≥ 100 × 10^9/L). Additionally, a clinically significant durable response (platelet count ≥ 30 × 10^9/L for at least 6 of the final 8 weeks of the core study) was noted in 64.0% of patients receiving avatrombopag, compared to 0% in the placebo group [55]. More than half (57.1%) of patients who had been on chronic corticosteroids experienced a dose reduction or discontinuation after initiating avatrombopag treatment. Safety profiles continued to support its use, with reduced bleeding episodes observed in the group treated with avatrombopag [53].

### 3.2 Real World Studies

The selection of TPO‐RAs for the management of ITP often hinges on factors such as pharmacy reimbursement policies. However, emerging real‐world evidence is gradually shaping clinical decisions by providing insights into the unique characteristics of the three currently approved TPO‐RAs (Table 2).

**TABLE 2:** Real world studies.

Study	Type	Patients enrolled (*n*)	Efficacy	Safety (AEs)
Oladapo A et al. REAL‐AVA 1.0 study [54]	Retrospective	Whole cohort (*n* = 205) Prior TPO‐RAs (6 months before initiating avatrombopag) *n* = 100 (49%) *n* = 51 (30%) Romiplostim *n* = 43 (25%) Eltrombopag *n* = 6 (6%) Both	Effectiveness subgroup (*n* = 21) 81% (*n* = 17) PC ≥ 30 × 10^9^/L 76% (*n* = 16) PC ≥ 50 × 10^9^/L 71% (*n* = 15) PC ≥ 75% × 10^9^/L 62% (*n* = 13) PC ≥ 100 × 10^9^/L	n.a.
GEPTI study [55]	Retrospective	Whole cohort (*n* = 268) Patients with baseline PC < 50 × 10^9^/L (*n* = 193) Patients with baseline PC ≥ 50 × 10^9^/L (*n* = 75)	Whole cohort versus patients PCs baseline < 50 × 10^9^/L Follow‐up since avatrombopag start (weeks), median (IQR): 47.5 versus 49.0 Response PCs ≥ 50 × 10^9^/L: 92% (247/268) versus 90.1% (174/193) Days to response, median (IQR): N.A. versus 13 LOR at the end of follow‐up: 3.3% (6/179) versus 4.9% (6/123)	Whole cohort (*n* = 268) No AEs 64% (*n* = 172) Thrombocytosis 14% (*n* = 37) Thrombocytosis leading to thrombosis 1% (*n* = 3) Thrombosis 3% (*n* = 9) Less severe AEs 17% (*n* = 47)
Al‐Samkari H. et al. study [56]	Retrospective	Whole cohort (*n* = 44) Mean ITP duration of 8.3 years Median (range) of four prior ITP treatments Prior TPO‐RAs: Romiplostim, ratio 33 (75%) Eltrombopag, ratio 10 (23%) Romiplostim/eltrombopag, ratio 1 (2%)	Whole cohort Platelet response (PC ≥ 50 × 10^9^/L): 93% (41/44) Complete response (PC ≥ 100 × 10^9^/L): 86% (38/44) Previous TPO‐RA patients Median PC 114 × 10^9^/L	n.a.

Abbreviations: GEPTI: Spanish ITP Group; n: number of patients; AEs: adverse events; TPO‐RAs: thrombopoietin receptor agonists; n.a.: not applicable; PC: platelet count; IQR: interquartile range; LOR, loss of response.

The REAL‐AVA 1.0 study was the first real‐world investigation of avatrombopag's use in patients with ITP, leveraging an administrative healthcare claims database linked to laboratory information. The patient cohort included patients with varying ages, comorbidity burdens, and histories of prior ITP treatments, with about half having used another TPO‐RA within six months before initiating avatrombopag. Out of 205 eligible patients, 49% reported prior TPO‐RA use. Notably, during follow‐up, approximately 70% and 93% of patients did not require rescue therapy with steroids or immunoglobulin, respectively. Among patients on concomitant steroids (*n* = 75) or immunosuppressants (*n* = 7) at avatrombopag initiation, 35% and 57%, respectively, discontinued these treatments. In the effectiveness subgroup (*n* = 21), 81% achieved clinically meaningful platelet count thresholds. Despite limitations related to the availability of platelet count data, these findings align with pivotal clinical trials suggesting avatrombopag's efficacy in real‐world practice [56].

The Spanish ITP Group (GEPTI) retrospective study further corroborated the effectiveness of avatrombopag across different patient profiles, including those with differing disease severity and treatment histories. The study encompassed 268 ITP patients newly treated with avatrombopag, with a median follow‐up duration of 47.5 weeks. Among the 193 patients with baseline platelet counts below 50 × 10^9/L, 90.1%, 174 patients achieved a platelet count of at least 50 × 10^9/L, and 87.6% 87.6% of the 129 patients who continued treatment maintained this threshold. Response was typically reached within 13 days (range 7–21 days). Even among patients with baseline platelet counts > 50 × 10^9/L, an impressive 97.3% responded, with 94.6% maintaining their response. Loss of response was consistently low (< 10%), regardless of ITP duration.

Among heavily pretreatment patients (> 4 prior treatment lines), 79% achieved desired platelet counts after switching to avatrombopag. Importantly, previous use of other TPO‐RAs (eltrombopag and/or romiplostim) did not affect the response rates, regardless of their prior success or failure. Avatrombopag enabled corticosteroid dose reduction or discontinuation in 80% of patients with low baseline platelet counts. AEs included thrombocytosis in 14.9% and thromboembolic events in 4.5% of patients [57].

A retrospective observational study by Al‐Samkari H. et al. evaluated avatrombopag in adults with ITP who transitioned from eltrombopag or romiplostim. Conducted across four U.S. tertiary ITP referral centers, the study included 44 patients with a mean ITP duration of 8.3 years and a median of four prior ITP treatments. Among these patients, 93% achieved a platelet response (≥ 50 × 10^9/L), and 86% yielded a complete response (≥ 100 × 10^9/L). The median platelet counts increased from 45 × 10^9/L on eltrombopag or romiplostim to 114 × 10^9/L after switching to avatrombopag (*p* < 0.0001). Specifically, for patients who switched due to inadequate response to romiplostim or eltrombopag, the median platelet count was 28 × 10^9/L before the switch compared to 88 × 10^9/L on avatrombopag (*p* = 0.025). Furthermore, 57% of patients on concomitant ITP medications, including 63% on chronic corticosteroids, discontinued these treatments after switching.

These findings highlight avatrombopag's ability to achieve high response rates, even among heavily pretreated chronic ITP population, with inadequate responses to previous TPO‐RAs [58].

These studies collectively underscore the utility of avatrombopag in real‐world settings, reinforcing its efficacy across diverse patient populations and treatment scenarios.

### 3.3 Ongoing Trials and Future Research

Ongoing research is poised to elucidate the broader applicability of avatrombopag across diverse patient demographics and clinical scenarios (Table 3).

**TABLE 3:** Ongoing clinical trials.

Study	Type phase	Population	Treatment	Scope
NCT04943042 (ADOPT trial)	Observational IV	ITP patients started on doptelet treatment (new users of Doptelet)	Avatrombopag	Prospective data: Usage, effectiveness, safety, patient‐ and clinician‐reported outcomes and health economic parameters Retrospective data: Collection of information on previous treatments, reason for treatment switch, healthcare resource use and, if applicable, Doptelet treatment prior to enrollment
NCT04638829	Prospective IV	cITP patients after switching to avatrombopag from eltrombopag or romiplostim: Total > 100 Prior eltrombopag 50 (±10) Prior romiplostim 50 (±10)	Avatrombopag oral tablet	Primary outcome measures: Safety and Tolerability (adverse events) of avatrombopag given for 90 days after stopping eltrombopag or romiplostim Secondary outcome measures: Subject reported outcomes Platelet counts
NCT01433978	III early terminated due to significant enrollment challenges	cITP patients: Splenectomized participants 35% of the study population No single PC is greater than 35 x 10^9/L Centrally stratified: Splenectomy status Baseline PC Use of concomitant ITP medication at baseline	Core study: 1:1 ratio:—avatrombopag—eltrombopag Extension Phase: Avatrombopag	Core study: To compare the efficacy of avatrombopag (in addition to standard of care) to eltrombopag (in addition to standard of care) for the treatment of adult participants with cITP as measured by durable platelet response Open‐label extension phase: To evaluate the safety and tolerability of long‐term therapy with avatrombopag participants with cITP

Abbreviations: cITP: chronic Immune Thrombocytopenia; PC: platelet count.

The ADOPT (NCT04943042) study is a Phase 4, observational, multicenter investigation designed to assess the real‐world effectiveness and utilization patterns of avatrombopag (marketed as Doptelet) in patients with ITP. The study includes both retrospective and prospective data collection. Retrospective data focuses on previous treatment history and healthcare resource use over the 12 months preceding avatrombopag initiation. Prospective data encompass drug usage, clinical effectiveness, safety, patient and clinician‐reported outcomes, and economic parameters. Eligible patients receiving avatrombopag will be monitored over 12 months (+ 6 months), with data gathered during clinical visits until the first appointment after one year or early study termination, whichever occurs first.

The NCT04638829 is a phase 4, prospective, multicenter, open‐label study that will evaluate the safety, platelet count, and subject‐reported medication satisfaction in adult subjects with chronic ITP after switching to avatrombopag from eltrombopag or romiplostim. At least 100 subjects will be enrolled, 50 (±10) who have received eltrombopag and 50 (±10) who have received romiplostim for at least 90 days prior to study entry.

The NCT01433978 is a phase 3, multicenter, randomized, double‐blind, active‐controlled, parallel‐group trial with an open‐label extension phase to evaluate the efficacy and safety of oral E5501 versus eltrombopag, in adults with chronic ITP. The study includes three phases: Pre‐randomization, Randomization (Core Study), and Extension Phase. Participants aged 18 and older who meet eligibility criteria will be randomized, ensuring at least 35% are splenectomized and no platelet count exceeds 35 × 10^9/L. Randomization will be centrally stratified by splenectomy status, baseline platelet count, and concomitant ITP medication, assigning participants to receive either double‐blind avatrombopag (20 mg daily, adjustable to 40 mg) or eltrombopag (50 mg daily, adjustable to 75 mg) in a 1:1 ratio. Dose modifications aim to maintain platelet counts between 50 × 10^9/L and 150 × 10^9/L and to reduce the need for additional ITP medications. The Core study lasts approximately 26 weeks, followed by an Extension Phase of about 104 weeks. However, this study is terminated early due to significant enrollment challenges.

The AVA‐PED‐301 study (NCT04516967) is a pivotal phase 3b trial aimed at assessing the efficacy and safety of avatrombopag in pediatric patients with ITP persisting for 6 months or longer. The trial enrolled 54 patients who were randomly assigned to receive either avatrombopag or a placebo in a 3:1 ratio for 12 weeks. Importantly, patients who completed this core treatment phase and met specific eligibility criteria were invited to continue in an open‐label extension phase lasting up to two years.

The results of this study, presented at the 2024 European Hematology Association (EHA) Congress, demonstrated the promising potential of avatrombopag in this population. The primary endpoint—durable platelet response, defined as a platelet count of at least 50 × 10^9/L without requiring rescue therapy for six or more weeks—was achieved by 28% of patients (95% CI, 15.8%–39.7%; *p* = 0.0077). Additionally, the key secondary endpoint, which measured the proportion of patients achieving two or more consecutive platelet counts ≥ 50 × 10^9/L without rescue therapy over at least six weeks, was met by 81.5% of participants (95% CI, 71.1%–91.8%; *p* < 0.0001).

Further analysis revealed several encouraging findings. By day 8, 56% of patients treated with avatrombopag reached platelet counts ≥ 50 × 10^9/L, compared to none in the placebo group (*p* < 0.0001). Moreover, the need for rescue therapy was required for markedly lower among avatrombopag recipients, with only 7% requiring additional treatment versus 43% in the placebo group (*p* = 0.0008). Safety data were equally reassuring; WHO grade 2 bleeding events occurred in 19% of patients in the avatrombopag group compared to 38% in the placebo group, and no thromboembolic events or deaths were reported during the core phase. Overall, 97% of patients transitioned into the open‐label extension phase, highlighting the feasibility of long‐term treatment with avatrombopag.

The clinical significance of these findings has drawn considerable attention, as avatrombopag appears poised to become an important treatment option for pediatric patients with persistent or chronic ITP. The FDA has recognized its potential and has set a target action date of July 24, 2025, for its approval in this indication [59, 60].

In parallel, the TX‐ITP‐001 (NCT06281327) is exploring a related, yet distinct, question regarding the use of avatrombopag in pediatric patients who have previously been treated with eltrombopag. This phase 2, is investigating the safety and efficacy of avatrombopag in children who may have experienced suboptimal efficacy, significant platelet fluctuations, or intolerance to eltrombopag, or who opted to switch due to personal preference, or economic considerations.

By addressing these nuanced aspects of avatrombopag use, this study aims to further clarify its role in pediatric ITP management and its potential to address unmet needs in this patient population.

Together, these studies underscore the growing evidence base for avatrombopag as a promising therapeutic option for pediatric ITP, with the potential to improve both efficacy and safety outcomes for this challenging condition.

## 4 Adverse Events

Avatrombopag has demonstrated a generally favorable safety profile in patients with chronic ITP, though ongoing surveillance of AEs remains essential as its use continues to expand.

Thromboembolic events occurred in 7% of patients (9/128) in pivotal trials, with 8 of these 9 patients presenting pre‐existing thrombotic risk factors. The rate aligns with safety data derived from long‐term studies examining romiplostim and eltrombopag, although the limited sample size warrants further investigation to better clarify the thrombotic risk related to avatrombopag. Common AEs reported include epistaxis, headache, fatigue, and confusion, with a similar incidence between avatrombopag and placebo groups in trials [52, 53]. Bleeding events were mild to moderate in 67% of patients during a Phase 2 trial [52], with serious events (WHO grade 3 or higher) occurring in only 3.1% of patients in a Phase 3 trial [53]. Liver function test elevations occurred in 5.5% of patients, resolving without discontinuation [61]. Unlike eltrombopag, avatrombopag has not demonstrated significant hepatotoxicity, eliminating the need for routine liver monitoring [40, 53]. Furthermore, in animal studies, the presence of avatrombopag in maternal milk and placental transfer raises concerns about its potential impact on fetal health if prescribed during pregnancy [62]. While animal studies did indicate a risk of pup mortality at much higher exposure levels than those in human patients, these findings underscore the importance of cautious assessment in pregnant populations [38]. Overall, while avatrombopag is generally well tolerated, careful monitoring for thromboembolic events, liver function, and other side effects is recommended in clinical practice.

Real‐world data from the US FDA's Adverse Event Reporting System (FAERS) from 2018 to 2023 complements clinical trial findings, revealing that among over 9 million cases, 1211 reported adverse events were “primarily suspected” to be associated with avatrombopag. The disproportionality analysis identified AEs such as decreased platelet counts (20.2%), headache (16.7%), and pulmonary embolism (2.3%), alongside unexpected AEs like seasonal allergies rhinorrhea, antiphospholipid syndrome, ear discomfort, and photopsia. Serious outcomes included hospitalization (34.6%), followed by death (15.4%). 64.2% of ITP patients experienced no AEs during treatment, reinforcing the overall safety of avatrombopag in managing ITP. Most AEs occurred within the first two days of initiating avatrombopag therapy, with a median onset time of 60 days reported for these events. During the initial month of treatment, AEs were notably reported on the first day (*n* = 57, 20.5%) and the second day (*n* = 35, 12.6%), after which the incidence of AEs began to stabilize. These findings underscore the importance of closely monitoring for avatrombopag‐associated AEs during the first month of treatment, particularly in the first two days following the administration of the medication, to maximize patient safety. Despite the limitations present in this study, it successfully identified new and unexpected AEs related to the clinical use of avatrombopag [63].

Furthermore, a recent meta‐analysis evaluating data from five randomized controlled trials comparing avatrombopag to placebo in patients with chronic thrombocytopenia, whether secondary to chronic liver disease or ITP, outlined criteria for severe adverse effects, which consisted of events such as thrombosis, acute myocardial infarction, and hypotension. Thrombosis was noted in only a few studies in this meta‐analysis, leaving the impact of avatrombopag on thrombosis unclear [64].

Additionally, long‐term safety data derived from recent studies, including a retrospective study conducted by the Spanish ITP Group (GEPTI) revealed that 40 out of 268 patients (14.9%) discontinued treatment upon achieving platelet counts exceeding 400 × 10^9/L, though only nine individuals did not resume therapy once their platelet count normalized. The study documented twelve cases of thrombosis (4.5%), none of which resulted in death. Notably, two cases of ITP were linked to antiphospholipid syndrome (APS), with one individual also diagnosed with systemic lupus erythematosus; these patients experienced acute myocardial infarction (AMI) and chronic inferior vena cava (IVC) thrombosis, underscoring the guideline recommendations against that give some suggestions to use these drugs with caution in this specific setting. Other documented events included three cases of ischemic stroke, two cases of pulmonary thromboembolism (PTE), one case of peripheral artery disease, and one case of AMI, with four patients resuming treatment before the study's conclusion. Mild and transient AEs were reported by 47 patients (17.5%), predominantly headaches (26 cases) and arthralgia (11 cases). Permanent discontinuation of avatrombopag due to AEs unrelated to thrombocytosis occurred in 12 patients (4.5%). Impressively, 172 out of 268 patients (64.2%) did not experience any AEs during their treatment with avatrombopag [55]. This real‐world evidence suggests that avatrombopag can be utilized safely for managing ITP, reaffirming previous findings [55, 56].

A case report published in February 2023 by Saartje Van de Vondel, MD, et al. detailed a patient with ITP who developed catastrophic antiphospholipid antibody syndrome (CAPS) following treatment with avatrombopag. While the direct causality between avatrombopag and the onset of CAPS in the absence of other risk factors remains uncertain, physicians need to be mindful of this potential risk. The use of TPO‐RAs in patients with antiphospholipid antibodies (APLA) who have ITP is not inherently contraindicated, particularly for those with a low to moderate thrombotic risk APLA profile. However, for patients identified as high‐risk, close monitoring is strongly advised. In this context, the objective of therapy shifts toward maintaining hemostatic platelet levels and preventing bleeding with minimal associated toxicity, rather than solely achieving platelet normalization [65].

The consistent reporting of mild AEs without significant hepatotoxicity distinguishes avatrombopag from other alternatives, as no cases of gastric atrophy or bone marrow pathology were noted. Preclinical studies have shown gastric toxicity at extremely high doses, yet clinical findings suggest a low risk of gastric toxicity at approved dosages [66]. Overall, while avatrombopag is generally well‐tolerated, thorough monitoring for thromboembolic events, liver function, and other AEs is crucial to enhance patient safety during clinical use.

## 5 New Clinical Indications

TPO‐RAs, including avatrombopag, have received regulatory approval in the US and Europe for treating thrombocytopenia associated with multiple conditions beyond ITP, such as chronic liver disease (CLD), severe aplastic anemia (SAA), and hepatitis C during antiviral therapy [30, 38, 40, 41]. CLD, which affected approximately 4.5 million adults in the US in 2018, frequently leads to thrombocytopenia in up to 90% of cirrhosis patients experiencing thrombocytopenia caused due to factors such as low TPO production and hypersplenism [67]. Traditional platelet transfusions, the standard for addressing periprocedural thrombocytopenia, have limitations, including short‐lived efficacy and transfusion‐related risks. Avatrombopag provides an effective alternative by enhancing platelet counts and minimizing transfusion requirements, without increasing thrombosis risk [68].

In SAA, characterized by T‐cell‐mediated bone marrow suppression and elevated interferon‐gamma (IFN‐γ) levels that disrupt TPO signaling, avatrombopag has shown therapeutic benefits. Similar to eltrombopag, avatrombopag binds to TPO receptors, offering a targeted mechanism of action to restore platelet counts [69].

Extensive clinical trials have demonstrated the effectiveness and safety of avatrombopag in managing thrombocytopenia unrelated to ITP. Current and ongoing studies aim to expand its therapeutic indications:

1. Periprocedural thrombocytopenia in CLD patients. A phase II study involving 130 patients revealed that avatrombopag significantly elevated platelet counts compared to placebo, with 47% achieving target platelet levels 4–8 days post‐treatment [54]. Subsequent Phase III trials (ADAPT‐1 and ADAPT‐2) confirmed decreased reliance on rescue procedures, reporting that up to 88% of avatrombopag‐treated patients avoided transfusions following surgery procedures [70]. These data were confirmed in Phase 4 observational study corroborated avatrombopag effectiveness in real‐world settings, further supporting its utility for this indication [71].
2. Chemotherapy‐induced thrombocytopenia (CIT). An open‐label trial with 74 patients showed that avatrombopag effectively increased platelet counts, achieving a 70.3% success rate at four weeks. Common AEs were generally mild, supporting the avatrombopag safety profile [72].

A case report highlighted the successful treatment of chemotherapy‐induced amegakaryocytic thrombocytopenia using a combination of avatrombopag and cyclosporine in a patient with recurrent ovarian cancer [73].

3 Non‐Severe Aplastic Anemia (NSAA). A phase II trial evaluated avatrombopag in 25 patients with refractory or intolerant NSAA. The study reported an 56% overall response rate at three months, suggesting that earlier treatment might improve short‐term outcomes. No serious AEs were reported [74].
4 Aplastic anemia secondary to chemo‐radiotherapy. A retrospective study of 34 patients with chemoradiotherapy‐induced aplastic anemia indicated a 58.8% overall response rate at six months. Higher cumulative doses of avatrombopag were associated with better responses [75].

These findings underscore avatrombopag's versatility for managing thrombocytopenia across various clinical conditions, including CLD, SAA, NSAA, and chemoradiotherapy‐induced aplastic anemia. The significant improvements in platelet counts and its favorable safety profile position avatrombopag as a promising therapeutic agent for expanding clinical applications.

## 6 Discussion

Avatrombopag has emerged as a valuable addition to the therapeutic arsenal for thrombocytopenia, particularly in ITP and other challenging conditions [37, 52, 53, 70, 71, 72, 73, 74, 75]. Its unique pharmacological profile—combining efficacy, safety, and patient‐centric features such as oral administration and the absence of dietary or hepatic toxicity constraints—addresses key limitations associated with older thrombopoietin receptor agonists (TPO‐RAs), having secured approval for use in the USA and Europe [40, 52, 53, 54, 55, 56, 57, 61].

Its standard initiation protocol involves a 20 mg daily oral dose, taken with food, to achieve and sustain platelet counts ≥ 50 × 10^9^/L. Initial weekly monitoring transitions to monthly assessments once platelet levels stabilize, with flexible dosing adjustments between 40 mg daily and 20 mg weekly based on individual responses. Treatment discontinuation is advised if no adequate response is achieved within four weeks at the maximum dose or if platelet counts exceed 400 × 10^9^/L on lower doses, followed by a four‐week observation period [37].

Current guidelines advocate TPO‐RAs as second‐line therapies for ITP. Although avatrombopag was not included in the ASH recommendations due to its regulatory status at the time, it is recognized in international consensus guidelines alongside romiplostim and eltrombopag [12, 13]. Selection among TPO‐RAs often hinges on reimbursement policies, but the increasing availability of real‐world data may refine treatment decisions based on specific pharmacological profiles [56, 57, 58].

Although avatrombopag is currently approved for use primarily in the chronic disease phase of ITP (duration > 12 months), emerging real‐world data suggest that it may also be effective in earlier stages of the disease. For instance, a Spanish study highlighted that the median time from ITP diagnosis or the initiation of first‐line treatment to avatrombopag was significantly shorter in patients over 65 years old, with a difference exceeding 12 months.

In cases where glucocorticosteroids, such as prednisone or dexamethasone, fail to quickly normalize platelet counts—especially in elderly patients on anticoagulation therapy—switching to second‐line treatments like TPO‐RAs is advisable. Rapid platelet recovery is crucial in such patients to minimize bleeding risk while avoiding the discontinuation of anticoagulants, as thrombocytopenia does not mitigate the risk of venous thromboembolism or stroke. Moreover, the study demonstrated that platelet response and the time required to achieve platelet recovery were unrelated to age, suggesting that avatrombopag is equally effective in both younger and older adult populations.

This further supports the use of avatrombopag in elderly and potentially frail patients, offering a means to normalize platelet counts efficiently while reducing prolonged exposure to immunosuppressive therapies. These findings position avatrombopag as a promising early intervention option in specific patient populations, particularly those requiring rapid platelet recovery with minimized immunosuppressive risks [58].

Direct comparative studies involving avatrombopag remain scarce. For instance, a trial comparing it to eltrombopag was terminated early due to recruitment difficulties. In place of direct comparisons, network meta‐analyses (NMAs) provide valuable insights, utilizing trial data with common comparators (e.g., placebo) to assess relative efficacy and safety among TPO‐RAs, including avatrombopag, eltrombopag, romiplostim, and fostamatinib [76].

Avatrombopag offers several distinct advantages over its counterparts. It lacks dietary restrictions and hepatotoxicity warnings, setting it apart from eltrombopag, and is administered orally, offering convenience over the subcutaneous delivery required by romiplostim. This aligns with the findings from the TRAPeze studies, a series of surveys conducted in Italy, UK, Ireland, and Netherlands, which investigated patient preference regarding TPO‐Ras. These studies revealed a clear preference among respondents for oral TPO‐RAs that require less frequent dosing and are free from dietary restrictions [77, 78, 79].

Furthermore, clinical evidence suggests its efficacy in refractory cases, with over 90% of patients who previously failed other therapies achieving platelet counts ≥ 50 × 10^9^/L upon transitioning to avatrombopag. These attributes position it as a compelling option, particularly for patients with concomitant liver disease.

Data on the outcomes of patients transitioning from romiplostim or eltrombopag to avatrombopag remain limited but promising. A retrospective observational study conducted across four US tertiary ITP referral centers reported that among adults with ITP who switched from eltrombopag to avatrombopag, 91% (10 out of 11) achieved both platelet response and complete platelet response. Additionally, of the five patients receiving concomitant corticosteroids during eltrombopag treatment, 40% (2 out of 5) were able to discontinue corticosteroids entirely, while the remaining 60% (3) successfully reduced their dosages. Importantly, no patients required the addition of new corticosteroid therapies while receiving avatrombopag treatment [58].

Avatrombopag has demonstrated high response rates comparable to other TPO receptor agonists. In the pivotal clinical trial, avatrombopag showed clear superiority over placebo, with 65.6% (21 out of 32) of patients achieving a platelet response by Day 8, compared to none of the 17 patients belonging to the placebo group (*p* < 0.0001). Real‐world data further support these findings, indicating rapid platelet recovery with avatrombopag treatment. In one study, patients with baseline platelet counts below 50 × 10^9/L reached a median response time of less than 15 days. The pivotal trial showed an even faster response, with 56% of patients achieving platelet counts of ≥ 30 × 10^9/L [53, 57]. Notably, all of these patients went on to normalize their platelet counts. Despite these encouraging results, no head‐to‐head studies directly comparing the different TPO‐Ras, including romiplostim, eltrombopag, and avatrombopag, have been conducted. Such studies would be valuable for clarifying potential differences in the speed and durability of platelet responses. Addressing this gap in evidence could significantly inform clinical decision‐making and optimize individualized treatment strategies for ITP. Future research in this area is eagerly anticipated.

The safety profile of avatrombopag is favorable, with minimal hepatic complications and no significant dietary constraints noted in clinical trials [51, 52, 53]. Although long‐term safety and effectiveness require further investigation, avatrombopag is increasingly viewed as an attractive therapeutic choice for cITP.

Thus, clinicians who previously faced challenges in managing thrombocytopenia across diverse patient populations now have the option to utilize avatrombopag. Its approval by both the FDA and EMA for the treatment of thrombocytopenia in adults with chronic liver disease undergoing medical or dental procedures provides a clear and evidence‐based pathway for its use in these specific clinical scenarios [54, 70, 71].

Clinical trial data and real‐world evidence consistently highlight its ability to achieve target platelet counts without increasing thrombotic risks. Furthermore, its lack of hepatotoxicity and the ability to bypass dietary restrictions position it as an ideal choice, particularly in patient subsets where eltrombopag or romiplostim might be less suitable.

From an economic perspective, avatrombopag stands out for its cost‐effectiveness, demonstrated in models that projected significant long‐term savings. In this respect, a budget impact analysis conducted for the Italian National Health Service (NHS) projected savings exceeding €6 million over three years with increased utilization, reinforcing its status as a cost‐efficient option for healthcare systems [77].

However, challenges remain. Comparative head‐to‐head trials are limited, and real‐world data, while promising, require further expansion to fully establish avatrombopag's positioning against other TPO‐RAs. Additionally, its role in newer indications, such as chemotherapy‐induced thrombocytopenia and aplastic anemia, warrants exploration in larger cohorts [72, 73, 74, 75]. These gaps in evidence, though narrowing, emphasize the need for ongoing research.

In summary, avatrombopag represents a well‐rounded and promising option in thrombocytopenia management. Its clinical and economic benefits are compelling, particularly for patients who have failed previous therapies or present with complex comorbidities. As data on long‐term outcomes and emerging indications continues to mature, avatrombopag is poised to play an increasingly central role in this field. For clinicians, its use should be tailored to individual patient profiles, ensuring optimal safety and efficacy while leveraging its unique advantages over existing alternatives.

## Author Contributions

All authors contributed to the manuscript and were involved in revisions and proofreading. All authors approved the submitted version.

## Conflicts of Interest

The authors declare no conflicts of interest.

## Data Availability

Data sharing is not applicable to this article as no new data were created or analyzed in this study.
